# Onset of Alpha-Gal Syndrome after Tick Bite, Washington, USA

**DOI:** 10.3201/eid3104.240577

**Published:** 2025-04

**Authors:** William K. Butler, Hanna N. Oltean, Elizabeth A. Dykstra, Eleanor Saunders, Johanna S. Salzer, Scott P. Commins

**Affiliations:** Kaiser Permanente Washington, Seattle, Washington, USA (W.K. Butler); Washington State Department of Health, Shoreline, Washington, USA (H.N. Oltean, E.A. Dykstra); Centers for Disease Control and Prevention, Atlanta, Georgia, USA (E. Saunders, J.S. Salzer); University of North Carolina School of Medicine, Chapel Hill, North Carolina, USA (E. Saunders, S.P. Commins)

**Keywords:** alpha-gal syndrome, anaphylaxis, tick bite, *Ixodes pacificus*, surveillance, parasites, vector-borne infections, zoonoses, Washington, United States

## Abstract

We describe a case of alpha-gal syndrome (AGS) in a resident of Washington, USA, after local *Ixodes pacificus* tick bites, which were associated with IgE increases after diagnosis. AGS should be considered a potential cause of anaphylactic and allergic reactions in persons with tick exposures, regardless of geographic residence.

Alpha-gal syndrome (AGS) is an allergy characterized by IgE-mediated hypersensitivity to galactose-α-1,3-galactose (α-gal), a disaccharide found in most nonprimate mammalian tissue (*1*). Epidemiologic studies have linked tick bites to AGS development (*2*–*5*). The number of documented AGS cases in the United States has increased since 2010, predominantly in the southern, midwestern, and mid-Atlantic regions (*6*). An association between lone star ticks (*Amblyomma americanum*) and α-gal sensitization has been well described. However, recent studies have identified cases outside the known range of this tick, and α-gal has been identified in the saliva of other tick species, including *Ixodes scapularis* (*6*,*7*). Evidence linking AGS to bites from ticks found in the western United States is lacking. We describe a case of AGS in a female resident of Washington, USA, who had symptom onset after tick bites in Washington and had no exposure to areas with known lone star ticks within the previous 30 years.

## The Study

In May 2017, a 61-year-old female wildlife biologist reported onset of diffuse urticaria and lip edema (day 0) (Figure). She called a consulting nurse and was advised to take ranitidine and diphenhydramine; symptoms resolved within 24 hours. On day 29, she had itching in her groin and noted urticaria on her back. Within a few minutes, she reported that her tongue felt swollen; she called 911, noting difficulty speaking. Paramedics documented diffuse urticaria, tongue edema, dysphonia, inspiratory wheezing, systolic blood pressure at 80 mm Hg, and heart rate at 120 beats/min. Epinephrine (0.3 mg) was administered intramuscularly (IM) for anaphylaxis, and mild improvement was observed. However, within several minutes, her clinical course worsened, and notable progression of tongue edema, throat tightness, and onset of tunnel vision occurred. A second dose of epinephrine (0.3 mg IM) was administered. While enroute to the emergency department, symptoms progressed, and she was given methylprednisolone (125 mg IM) and nebulized racemic epinephrine. Upon arrival at the emergency department, her symptoms were improving, and examination revealed mild urticaria and blood pressure of 117/57 mm Hg. She was discharged after a 4-hour observation and prescribed prednisone, famotidine, diphenhydramine, and an epinephrine autoinjector.

**Figure:**
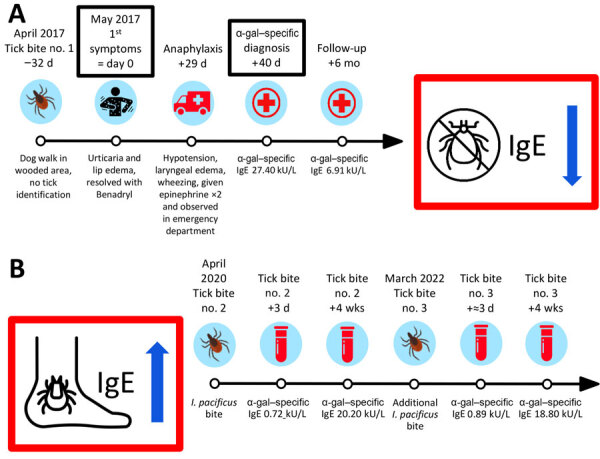
Timeline of tick bite exposures, symptoms, healthcare visits, and laboratory results for patient in study of alpha-gal syndrome, Washington, USA, 2017–2022. A) Timeline after first tick bite in 2017. B) Timeline of second (2020) and third (2022) bites from *Ixodes pacificus* ticks.

No unusual activities or exposures immediately preceding either episode (day 0 or 29) were documented in the initial exposure history, although she was outside before both reactions. She had no known stings immediately preceding symptoms and no previous illnesses. She was not taking routine medications and reported no previous episodes of hives or swelling. During a later interview, the patient recalled that ≈1 month before the initial episode (day −32), she had walked her dog in a wooded area and had itching on her back. She had inspected the area and found a nonengorged tick embedded in her left shoulder, estimating ≈12 hours of attachment. She had attempted to remove the tick, but some mouthpart material remained; the area subsequently became painful and red, enlarging to a size greater than the palm of her hand. She sought care at the emergency department the same day, where mouthparts were removed and doxycycline was prescribed.

A detailed food history indicated the patient had eaten beef tacos 6 hours before the first episode of angioedema (day 0) and consumed both pork sausage 9 hours and a ham sandwich 4 hours before the second reaction (day 29); she reported rarely eating red meat. She recalled no other tick bites since working in Wyoming in the 1980s. Her lifetime travel history included brief visits to Florida and Maryland (lone star tick populations expected) >30 years before, as well as a trip to northern Illinois in 2016 along with a road trip back to Washington through Minnesota, South Dakota, Montana, and Idaho (lone star tick populations not established).

On day 40, an allergist (W.K.B.) evaluated her for presumed idiopathic anaphylaxis. The following laboratory results were within reference ranges: complete blood cell count, Chronic Urticaria Index (4.8 units), serum protein electrophoresis, bee venom–specific IgE (<1 ng/mL for 6 stinging insect venoms), tryptase (5.4 ng/mL), and 24-hour N-methylhistamine (129 μg/g creatinine). However, her α-gal IgE was positive at 27.40 kU/L (reference range <0.1 kU/L). AGS was diagnosed, and she was told to strictly avoid consuming mammalian meat and cautioned about consuming milk and gelatin. She subsequently avoided meat and gelatin continuously and had no further allergic episodes. Within 6 months, her α-gal IgE had fallen from 27.40 kU/L to 6.91 kU/L in the absence of intervening tick bites.

In April 2020, the patient reported a second tick bite that occurred ≈1 mile from the original bite in 2017. The tick was embedded on her upper back for ≈10 hours before removal. Her cutaneous reaction to this bite site was similar to that of the previous episode (redness, heat, and extreme itchiness), and the bite area measured ≈1.5 inches in diameter. Three days after this second bite, her α-gal IgE level was 0.72 kU/L, increasing to 20.20 kU/L 4 weeks later. She submitted the tick to the Washington State Department of Health through the passive tick surveillance program, where it was identified as an adult female *I. pacificus* tick. Had the patient not switched to a vegetarian diet, clinical experience suggests that the rise in IgE titer might have increased her chances of having an allergic reaction after red meat consumption (*8*).

In March 2022, the patient reported a third tick bite on the inside of her right knee. Tick attachment was estimated to be <1 hour. She experienced a similar itchy reaction at the bite site. Clinical follow-up documented another IgE titer increase, from 0.89 kU/L initially to 18.80 kU/L 4 weeks later. She also submitted this tick to the Washington State Department of Health, where it was identified as an adult female *I. pacificus* tick*.* The patient included details regarding her symptoms and AGS diagnosis with this tick submission, and the Washington State Department of Health initiated a public health investigation, which included a detailed exposure interview.

The habitat of the *I. pacificus* tick is known to extend along the Pacific Coast of the United States; it is commonly found in northern California, Oregon, and Washington and the coast of British Columbia, Canada (*9*). Although the genus *Ixodes* has been associated with AGS in Australia (*10*), Scandinavia (*11*), and Europe (*12*–*14*), it has yet to be conclusively linked with AGS in the United States. Commercial laboratories have documented α-gal–specific IgE in samples from patients residing outside of the known range of lone star ticks, including in several western US states (*6*,*15*). Although those persons might have traveled, the possibility of local exposure cannot be excluded. However, observed AGS incidence in those areas appears low relative to the high number of reported *Ixodes* tick bites. Therefore, those findings might reflect a limited role for tick species other than *A. americanum* in AGS development in the United States, which is further supported through review of geographic distribution of suspected cases and range of *A. amblyomma* ticks (*6*,*15*).

## Conclusions

We provide evidence that bites from *I. pacificus* ticks might stimulate an IgE response leading to AGS, particularly after repeated tick bites. In addition, we highlight the value of passive tick surveillance programs, which led to public health reporting of this uncommon finding. Additional work will be needed to determine a possible link between *I. pacificus* or other *Ixodes* spp. ticks and AGS in the United States. Providers should consider AGS as a cause of anaphylaxis in the western United States. Public health practitioners across the United States should continue efforts focused on tick bite prevention, healthcare provider education, and improved tick and tickborne disease surveillance.
